# Association Between Ellipsoid Zone Area and Fixation Stability in Macular Telangiectasia Type 2

**DOI:** 10.1167/iovs.67.12.57

**Published:** 2026-10-23

**Authors:** Kensington A. Hatcher, Thomas M. Aaberg, Joanna Yankey, Eleonora M. Lad, Razek G. Coussa, Robert Kissling, Muna I. Bitar

**Affiliations:** 1 Neurotech Pharmaceuticals, Cumberland, Rhode Island, United States; 2 Foundation for Vision Research, Grand Rapids, Michigan, United States; 3 Veristat, Southborough, Massachusetts, United States; 4 Department of Ophthalmology, Duke University Medical Center, Durham, North Carolina, United States; 5 Retina Vitreous Consultants of Wisconsin, Milwaukee, Wisconsin, United States

**Keywords:** ellipsoid zone area loss, fixation stability, macular telangiectasia type 2, retrospective longitudinal analysis, revakinagene taroretcel-lwey

## Abstract

**Purpose:**

To determine whether ellipsoid zone (EZ) area loss, a biomarker of photoreceptor degeneration, is associated with fixation stability in macular telangiectasia type 2 (MacTel).

**Methods:**

This retrospective analysis included 212 adults from two Phase 3 trials of revakinagene taroretcel-lwey for MacTel. EZ area loss was quantified with optical coherence tomography. Fixation stability (P1 index) was measured by microperimetry. Linear and logistic regressions evaluated associations between EZ area loss and P1 over 24 months. A median split stratified the population to evaluate whether baseline EZ area loss influenced the therapeutic effect of the implant versus sham. A receiver operating characteristic analysis identified a structural threshold for unstable fixation.

**Results:**

EZ area loss was significantly associated with reduced fixation stability at baseline (*P* = 0.002) and month 24 (*P* = 0.01). Each 1-mm² increase in EZ area loss doubled the odds of poor fixation stability (defined as a P1 <80%) (odds ratio, 2.07; *P* = 0.047). P1 change did not significantly differ between the treatment and sham groups. In exploratory analyses, stratification by median baseline EZ area loss, despite a nonsignificant treatment-by-baseline interaction (*P* = 0.17), revealed a greater decline in fixation stability in sham-treated eyes (mean, −3.8%) versus the implant group (+0.6%). The receiver operating characteristic analysis identified an EZ area loss threshold of 1.075 mm² for unstable fixation.

**Conclusions:**

Greater EZ area loss is associated with poorer fixation stability in MacTel. An EZ area loss threshold of ∼1 mm² may serve as an exploratory marker of fixation instability risk, though this threshold requires independent validation.

Macular telangiectasia type 2 (MacTel) is a bilateral, neurodegenerative disease characterized by photoreceptor loss, Müller cell dysfunction, and right-angle vascular changes.^1^^,^^2^ Progressive disruption of the photoreceptor layer contributes to declining visual function, including impaired reading, metamorphopsia, and difficulty maintaining a stable preferred retinal locus (PRL).^3^^–^^5^

The ellipsoid zone (EZ), representing mitochondria-dense photoreceptor inner segments, is reliably visualized using spectral-domain optical coherence tomography.^6^ Loss of EZ area serves as a structural biomarker of photoreceptor degeneration in MacTel and has been correlated with multiple measures of visual function, including retinal sensitivity.^7^^,^^8^ Although these findings establish a relationship between EZ degeneration and localized retinal function, retinal sensitivity and fixation stability represent distinct aspects of visual performance. Retinal sensitivity quantifies light detection at individual retinal locations, whereas fixation stability reflects the ability to maintain the line of sight on a target and is influenced by the integrity and functional use of the PRL.

Fixation stability, measured using microperimetry, is a clinically meaningful indicator of macular function and is relevant to the functional use of the PRL.^5^^,^^9^ The Macular Integrity Assessment (MAIA) P1 index, defined as the percentage of fixation points within 1° of the target, provides a quantitative measure of central fixation containment. In MacTel, where progressive parafoveal neurodegeneration and scotoma development can disrupt the PRL, fixation stability may provide functional information that is not captured by retinal sensitivity alone.^10^^–^^12^ However, the relationship between quantitative EZ area loss and fixation stability has not been systematically characterized in MacTel.

The purpose of this study was therefore to evaluate the association between EZ area loss and fixation stability at baseline and over 24 months, as well as to characterize longitudinal structural progression, in a well-characterized cohort of adults with MacTel.

## Methods

### Study Design

This retrospective longitudinal analysis utilized data from NTMT-03-A (NCT03316300) and NTMT-03-B (NCT03319849), two pivotal phase 3 trials of revakinagene taroretcel-lwey (Neurotech Pharmaceuticals, Cumberland, RI, USA) for MacTel.^13^ These studies adhered to the tenets of the Declaration of Helsinki and were approved by the institutional review boards of each participating center.^13^ Informed consent was obtained from all participants.^13^

One eye per participant was included in this analysis. Participant eyes were included if both EZ area loss measurements and microperimetry data were available at baseline and 24 months. Eyes with incomplete structural or functional data were excluded from the analysis. EZ area loss was quantified using en face spectral-domain optical coherence tomography images by the Duke Reading Center (Durham, NC, USA) by readers masked to treatment allocation. Full methodological details regarding image acquisition and grading are available in the primary phase 3 trial publication.^13^ Fixation stability was measured with the MAIA microperimeter (CenterVue, Padova, Italy) under standardized mesopic testing conditions using a custom 85-loci stimulus grid centered on the fovea.^14^ The P1 fixation stability index (percentage) served as the primary endpoint. The P1 fixation stability index measures how often the eye remains within ±1° of the fixation target during microperimetry testing.^10^ Fixation losses (percentage) were recorded as a reliability covariate.

### Statistical Analyses

Multiple linear regression models were fitted independently at baseline and month 24 to evaluate EZ area loss (mm^2^) as a continuous predictor of P1 fixation stability, best-corrected visual acuity (BCVA), and reading speed, controlling for age and fixation losses. Model residuals and homoscedasticity assumptions were formally evaluated using residual versus fitted plots, normal Q–Q plots, and the Breusch–Pagan test. To assess consistency across clinical trials, separate multivariable models were evaluated for study A and study B, and a formal interaction model incorporating a trial term (baseline EZ area loss × trial) was performed. Logistic regression was used to evaluate predictors of poor fixation stability (P1 <80%).

Longitudinal trajectories were evaluated using linear mixed-effects models incorporating subject-level random intercepts and fixed effects for study visit, treatment arm, baseline EZ area loss, age, and their interactions, estimated via restricted maximum likelihood with Satterthwaite's degrees of freedom.

To exploratorily evaluate whether treatment efficacy varied according to baseline disease severity, a formal interaction term between treatment group and baseline EZ area loss was incorporated into the mixed-effects models. To visualize these exploratory subgroup dynamics, the cohort was secondarily stratified using the cohort median baseline EZ area loss threshold (0.38 mm^2^), and group-specific least squares mean changes with 95% confidence intervals were estimated using estimated marginal means.

Receiver operating characteristic (ROC) curve analyses were performed to determine structural thresholds predictive of unstable fixation. A secondary sensitivity analysis was conducted using the log-transformed 95% bivariate contour ellipse area (BCEA) to verify primary structural–functional relationships. Statistical significance was defined as *P* < 0.05, and all statistical analyses were performed using R statistical software (version 4.4.2; R Project for Statistical Computing, Vienna, Austria).

## Results

### Study Population

Of the 228 participants enrolled, 212 (93%) had complete baseline and 24-month data and were included in the longitudinal analysis. The mean (standard deviation) age at baseline was 59.6 (8.2) years, and 149 (70.3%) participants were female. The mean (standard deviation) EZ area loss at baseline was 0.49 (0.37) mm^2^. Baseline characteristics of the analyzed cohort, stratified by EZ area loss group and treatment arm, are summarized in the Table. Mean age was similar across treatment arms for both EZ area loss groups. Baseline P1 fixation stability was nearly identical between treatment arms in the low EZ area loss group (P1 in implant group = 87.3%; P1 in sham group = 87.5%). In the high EZ area loss group, baseline P1 fixation stability was numerically higher in the sham group (P1 = 84.7%) than in the implant group (P1 = 80.6%).

**Table.** Baseline Demographics and Clinical Characteristics in Participants With Low EZ Area Loss and High EZ Area Loss

	Low EZ Area Loss (*n* = 106)		High EZ Area Loss (*n* = 106)	
Variable	Implant (*n* = 51)	Sham (*n* = 55)	Implant (*n* = 58)	Sham (*n* = 48)
Age, mean ± SD, y	60.7 ± 7.78	58.9 ± 8.77	58.8 ± 7.76	59.6 ± 8.43
Baseline EZ area loss, mean ± SD, mm^2^	0.25 ± 0.068	0.25 ± 0.067	0.76 ± 0.417	0.71 ± 0.332
Baseline P1 fixation stability, mean ± SD, %	87.3 ± 14.8	87.5 ± 15.2	80.6 ± 20.4	84.7 ± 18.1

The low EZ area loss group included participants with baseline EZ area loss < 0.38 mm^2^, and the high EZ area loss group included those with baseline EZ area loss ≥ 0.38 mm^2^.

### Association Between EZ Area Loss and P1 Fixation Stability

The multivariate regression analysis identified baseline EZ area loss as significantly associated with reduced fixation stability. At baseline, every 1-mm^2^ increase in EZ area loss was associated with a 9.42% decrease in P1 fixation stability (β = −9.42; standard error = 3.06; *P* = 0.002). This association persisted longitudinally, and the relationship remained statistically significant (β = −7.89; standard error = 3.05; *P* = 0.01) at the 24-month follow-up. Heatmaps highlight the high-concentration “core” of the patient population, demonstrating a clear downward trend in fixation stability as EZ area loss increases (Fig. 1); this visualization emphasizes the primary biological signal while accounting for the high degree of clinical variability inherent in microperimetry.

**Figure 1.**
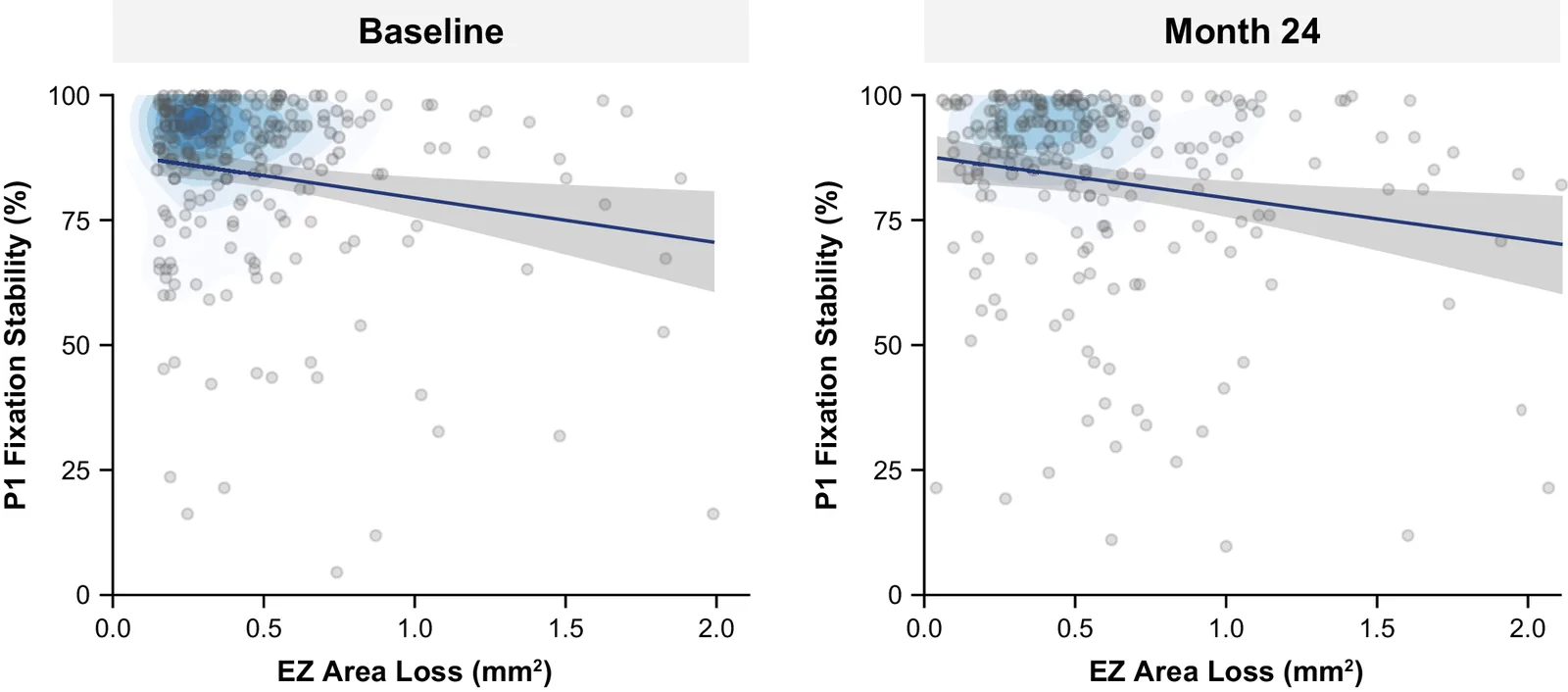
Density mapping of a structure‒function relationship. This scatterplot shows the relationship between EZ area loss (mm^2^) and P1 fixation stability (percentage) at baseline and month 24, with overlaid density contours generated via two-dimensional kernel density estimation. The regression lines (*solid*) and 95% confidence intervals (*shaded*) illustrate the significant decline in fixation stability as the EZ area loss increases.

### Evaluation of Model Diagnostics and Robustness

Model diagnostics indicated mild heteroscedasticity (Breusch–Pagan *P* = 0.032). Because sensitivity analyses using HC3 robust standard errors yielded identical statistical significance, standard ordinary least squares models are reported.

### Factors Associated With Poor P1 Fixation Stability

The multivariate logistic regression demonstrated that among the three variables evaluated (i.e., EZ area loss at month 24, fixation loss at month 24, participant age at enrollment), EZ area loss has the greatest association with poor fixation stability (P1 < 80%). Each 1-mm² increase in EZ area loss approximately doubled the odds of poor fixation (odds ratio [OR] = 2.07, *P* = 0.047). This relationship remained significant after adjusting for participant age (OR = 1.04, *P* = 0.042) and fixation losses (OR = 1.03, *P* = 0.030).

### Longitudinal Structural Progression (EZ Area)

The Linear mixed model (LMM) revealed a significant effect of time on the total area of EZ area loss (*P* < 0.001). Participants categorized in the cohort with high EZ area loss at baseline (≥0.38 mm^2^) demonstrated a significantly accelerated rate of further EZ area loss compared with the group with low EZ area loss at baseline (<0.38 mm^2^; group × time interaction; *P* = 0.009). Over the 24-month study, the mean ± standard deviation EZ area loss in the high EZ area loss group expanded from 0.74 ± 0.4 to 0.90 ± 0.4 mm^2^. In contrast, the low EZ area loss group exhibited slower progression, with mean EZ area loss increasing from 0.25 ± 0.1 to 0.35 ± 0.2 mm^2^.

### Longitudinal Functional Impact (P1 Fixation Stability)

At baseline, no statistically significant difference was observed in P1 fixation stability between the high EZ area loss and low EZ area loss groups (*P* = 0.232). Eyes with greater EZ area lesion size at baseline showed numerically lower percent fixation stability at 24 months in the LMM compared with those with less than the median loss, but this difference was not statistically significant (*P* = 0.084) (Fig. 2).

**Figure 2.**
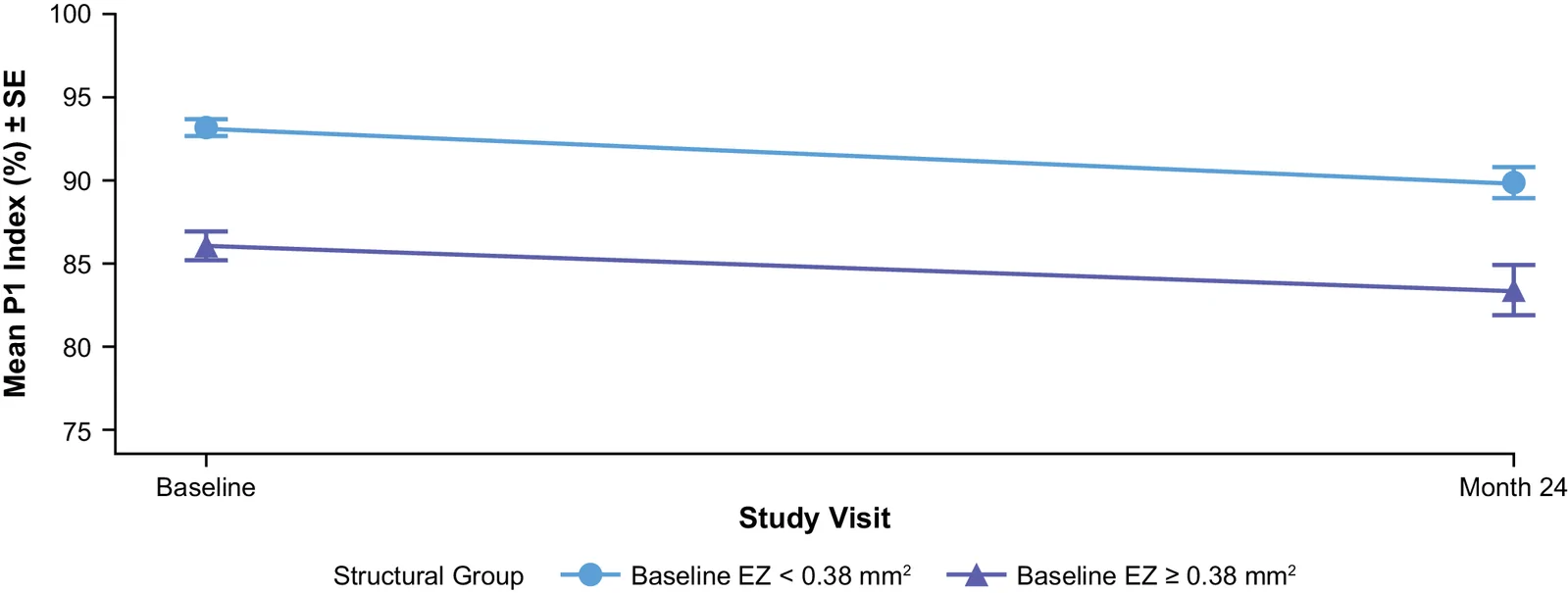
Longitudinal fixation stability by baseline EZ area loss group. The line graph displays the mean P1 index (percentage) at baseline and 24 months for two treatment cohorts: those with baseline EZ area loss <0.38 mm^2^ (*blue line*) and those with baseline EZ area loss ≥0.38 mm^2^ (*purple line*). *Error bars* represent the standard error (SE).

### Treatment Response Stratified by Baseline EZ Area Loss

In the total longitudinal cohort, the difference in mean P1 change between the implant and sham surgery did not reach statistical significance at 24 months (−0.8% vs. −2.6%; *P* = 0.45). The formal interaction between treatment assignment and continuous baseline EZ area loss was also not statistically significant (*P* = 0.17), providing no evidence that treatment efficacy differed according to baseline EZ area loss. In an exploratory, descriptive analysis, the cohort was stratified by the median baseline EZ area loss. In the low EZ area loss group, both implant and sham surgery arms maintained high functional stability throughout the 24-month study. In the high EZ area loss group, sham-treated eyes had a mean P1 change of −3.8%, whereas implant-treated eyes had a mean change of +0.6%; however, the between-treatment comparison at month 24 was not statistically significant (*P* = 0.90) (Fig. 3). These median-split findings are therefore considered hypothesis-generating rather than evidence of differential treatment efficacy.

**Figure 3.**
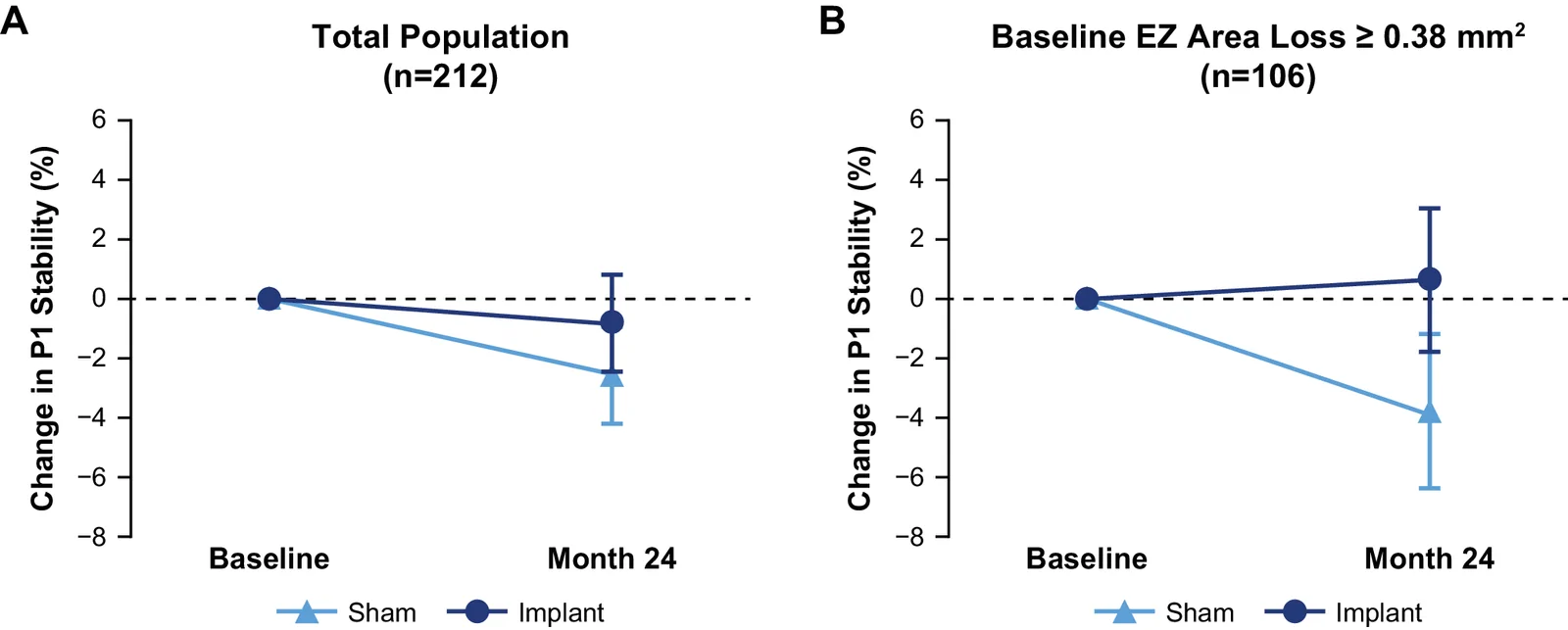
Response to treatment based on baseline structural severity. Change in P1 fixation stability (percentage) from baseline to 24 months for the (**A**) total cohort and (**B**) eyes exceeding the median structural threshold. *Error bars* represent the standard error of the mean. The *horizontal dashed line* at zero represents the baseline functional state; data points below this line indicate a loss of fixation stability, while points on or above the line indicate functional preservation or improvement. In the total population (*N* = 212) graph, a numerical trend favoring the revakinagene taroretcel-lwey implant over sham surgery was observed (mean change, −0.8% vs. −2.6%); however, the difference was not statistically significant (*P* = 0.45). In the baseline EZ area loss ≥0.38 mm^2^ graph (*N* = 106), stratification by median baseline EZ area loss reveals a divergence in trajectories. While sham-treated eyes exhibited rapid functional decline (mean change, −3.8%), eyes receiving the implant achieved functional stabilization (mean change, +0.6%).

### Receiver Operating Characteristic Threshold

The multivariate model for predicting poor fixation stability based on EZ area loss, adjusting for age and fixation losses, showed fair discriminative ability (area under the curve [AUC], 0.66). Using the Youden index, the optimal probability threshold for predicting poor fixation was identified as 0.307. At this cutoff, the model achieved a high specificity of 0.835 but a limited sensitivity of 0.426. To translate these probabilities into a clinically meaningful measure, we solved the regression equation for EZ area loss while holding covariates at their cohort means (age, 59.6 years; fixation losses, 7.20). This equation identified an EZ area loss of 1.075 mm^2^ as the structural threshold for increased risk of poor fixation stability (Fig. 4).

**Figure 4.**
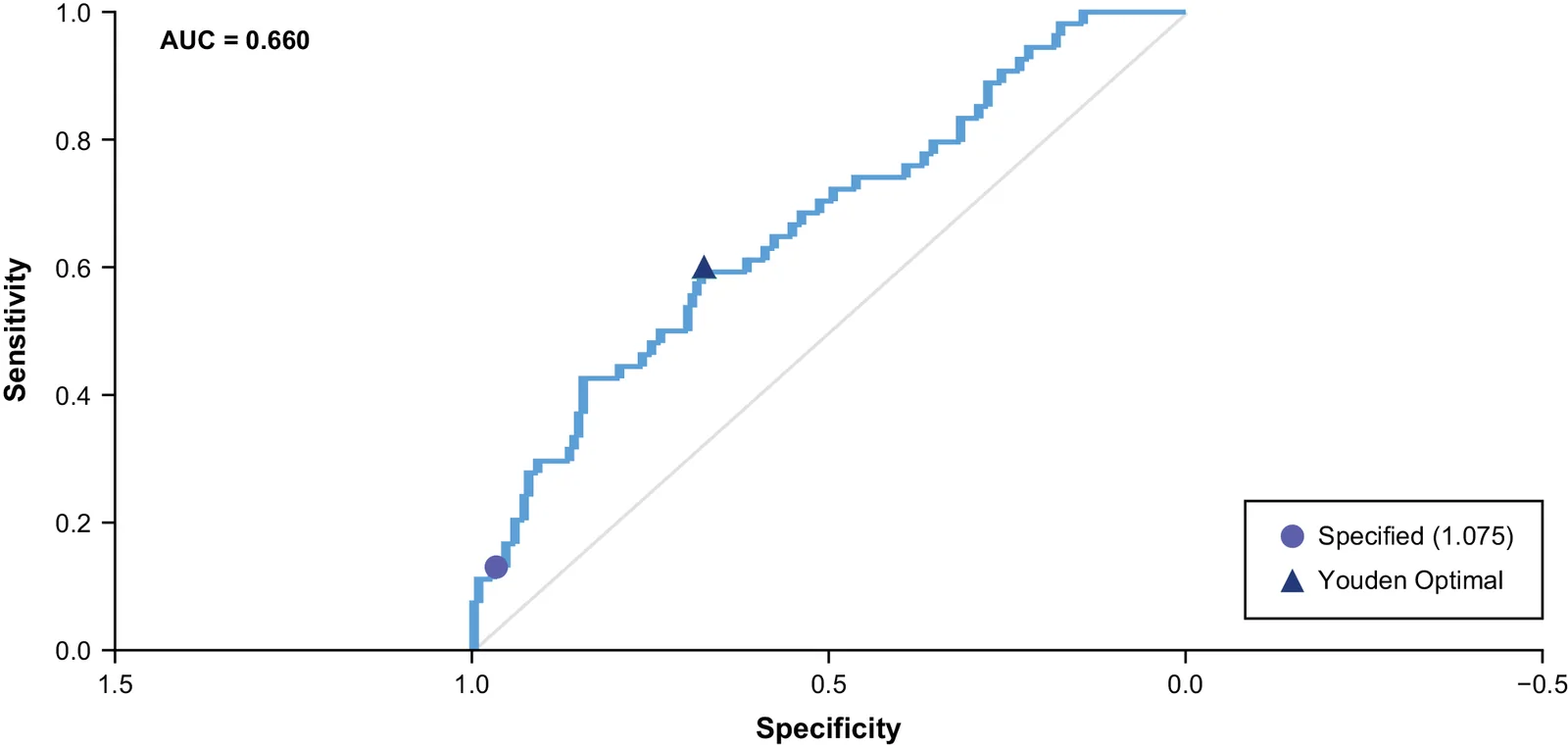
ROC curve: EZ area loss as a marker of unstable fixation. The ROC curve for EZ area loss is a marker of unstable fixation. The ROC curve (*light blue line*) illustrates the diagnostic ability of EZ area loss to discriminate between participants who developed unstable fixation versus those who did not over the 24 months. The AUC is 0.660, indicating a modest predictive value. The *dark blue triangle* represents the Youden optimal cutoff points, which maximize the sum of sensitivity and specificity. The *purple circle* indicates a threshold of 1.075 mm^2^. The diagonal gray line represents the line of identity (AUC, 0.50), corresponding to the predictor with no better than random chance performance.

### Sensitivity Analysis of Fixation Stability (BCEA95%)

Substituting BCEA95% for the P1 index confirmed the primary structure–functional relationships observed in this cohort. In the multivariable linear regression model, greater baseline EZ area loss was significantly associated with an expanded BCEA95% ellipse area (b = 0.21, SE = 0.09, *P* = 0.013). The ROC curve analysis for the BCEA95% instability model yielded an AUC of 0.72, indicating acceptable discrimination performance and demonstrating that the total spatial dispersion captures the functional consequences of outer retinal degeneration.

### Trial-Stratified Analysis

To explore whether trial-specific reading speed differences (noted in the parent studies for NTMT-03-A versus NTMT-03-B) may relate to fixation stability, trial-stratified models were evaluated. While baseline associations varied slightly by cohort, the EZ-by-trial interaction was nonsignificant (*P* = 0.126), indicating comparable structure–function coupling across trials.

Baseline P1 fixation stability did not predict subsequent change in reading speed in the overall cohort (β = −0.14, *P* = 0.346), and the relationship between baseline P1 and change in reading speed did not differ between NTMT-03-A and NTMT-03-B (P1-by-trial interaction: β = −0.18, *P* = 0.533). Results were consistent when fixation stability was quantified using BCEA. Baseline log-transformed BCEA was not associated with change in reading speed (*P* = 0.616), and the BCEA-by-trial interaction was not significant (*P* = 0.297).

### Relationship Between Fixation Stability and Visual Acuity

Baseline P1 fixation stability was associated with baseline BCVA after adjustment for baseline EZ loss and age (β = 0.10 Early Treatment Diabetic Retinopathy Study letters per 1% increase in P1, *P* = 0.002). However, baseline P1 did not predict change in BCVA over 24 months (β = −0.004, *P* = 0.860). Results were consistent when analyses were restricted to sham-treated eyes: baseline P1 did not predict subsequent BCVA change (β = 0.038, *P* = 0.253), and change in P1 was not associated with change in BCVA (β = −0.020, *P* = 0.505). Similarly, baseline BCEA was not associated with subsequent BCVA sham in sham-treated eyes (β = −0.99, *P* = 0.360).

## Discussion

This retrospective longitudinal analysis of two phase 3 studies demonstrates that EZ area loss is significantly associated with fixation instability in MacTel, reinforcing the central role of photoreceptor structural integrity in visual function.^4^^,^^13^ The strong association between EZ area loss and fixation performance aligns with prior work demonstrating a structural–functional relationship. For example, Mukherjee et al.^15^ reported a nearly perfect correlation (*r* ≈ 0.93–0.94) between EZ area loss size and retinal sensitivity measured by MAIA microperimetry, highlighting EZ disruption as a key determinant of localized functional vision loss.

Longitudinal data from the MacTel Project further support this relationship. Heeren et al.^16^ showed that progression of EZ area loss closely paralleled scotoma enlargement, reinforcing EZ area loss as a sensitive marker of neurodegenerative progression. Other multimodal imaging studies also corroborate this relationship. Ledolter et al.^17^ identified EZ area loss as the strongest predictor of sensitivity reductions among multiple optical coherence tomography biomarkers, while Tzaridis and Friedlander^18^ reported that both EZ area loss and hyperreflective lesion area were important topographic predictors of functional deficits. Collectively, these studies establish a robust foundation for the finding that EZ integrity underlies retinal sensitivity.

This analysis extends these prior works in several important ways. Whereas previous studies emphasized retinal sensitivity and scotoma formation, fixation stability, a key determinant of reading performance and daily visual function, has been less thoroughly investigated in MacTel. Our logistic regression analysis quantifies this risk, revealing that for every 1-mm^2^ increase in EZ area loss, the odds of falling below the 80% fixation stability threshold (P1) more than double. By demonstrating that EZ area loss is significantly associated with fixation instability after adjusting for age and fixation losses, this current study broadens the functional implications of EZ disruption beyond sensitivity measures alone.

The exploratory median-split analysis provides descriptive observations regarding fixation stability across baseline EZ area loss strata but does not establish differential treatment efficacy. Although the high EZ area loss subgroup showed a numerically different trajectory between treatment groups, the between-treatment comparison at month 24 was not statistically significant, and the formal treatment × continuous baseline EZ area loss interaction was also not statistically significant. Accordingly, these findings should be interpreted as hypothesis-generating rather than as evidence that treatment efficacy varies according to baseline EZ area loss. The potential for structural disease severity to influence detectability of functional treatment effects has also been recognized in geographic atrophy and other retinal degeneration trials, where functional measures may lag behind structural changes.^19^^,^^20^ These exploratory subgroup observations align with the consensus that structural markers, such as EZ area loss, provide more sensitive endpoints for assessing disease progression and underscore that early intervention may optimize long-term outcomes in slowly progressive neurodegenerative diseases.^21^

In this analysis, we defined stable fixation as a P1 index ≥80%, a threshold intentionally more stringent than the ≥75% cutoff commonly used to classify fixation stability traditionally in MAIA microperimetry.^10^^,^^22^^,^^23^ This tighter criterion was selected as a quality control measure to reduce fixation-related spatial variability and to ensure that structure‒function correlations were derived from measurements obtained under highly centered fixation conditions. Such precision is particularly important in MacTel, where EZ area loss generally involves parafoveal and foveal regions, making accurate foveal centration critical for meaningful spatial alignment of sensitivity data.^7^ Additionally, MAIA testing in these trials employed a custom 85-loci grid centered on the fovea; therefore, even subtle micro-instabilities could introduce variability in pointwise sensitivity mapping.^15^ Adopting a stricter threshold was intended to mitigate this potential noise and enhance the robustness of these analyses rather than to redefine fixation stability classification.

Although BCEA is a widely recognized metric for quantifying fixation dispersion, it characterizes the spatial distribution of fixation points, whereas the P1 index quantifies the proportion of fixation points maintained within 1° of the PRL.^10^^,^^15^ Because our analysis specifically examined the relationship between EZ area loss and centrally maintained fixation, we selected P1 as the primary measure of fixation stability. P1 provides a clinically intuitive measure of central fixation containment within 1° of the PRL.^10^ P2 was excluded due to lower sensitivity and ceiling effects, while BCEA was evaluated as a secondary measure in sensitivity analyses. Accordingly, P1 was selected as the primary fixation metric because it was most closely aligned with the objectives of the present study, while BCEA was evaluated as a complementary measure in a sensitivity analysis.

Our results complement findings from MacTel Project Report No. 6, which showed that foveal EZ area loss predicts visual acuity decline over time, underscoring that photoreceptor preservation is critical for maintaining multiple aspects of visual function.^8^ Together, these studies emphasize the importance of EZ integrity in supporting functional vision, including visual acuity, retinal sensitivity, and now fixation stability.

An exploratory finding of this analysis is the identification of a structural threshold of approximately 1 mm² of EZ area loss. Individuals below this threshold generally maintain reliable fixation, reflecting the high specificity in the model. A loss exceeding 1 mm^2^ does not automatically indicate poor fixation (due to lower sensitivity) but does identify a high-risk group that may require closer monitoring, repeat testing, or additional functional assessment. This interpretation is consistent with photoreceptor sampling models and fixation studies in retinal disease, which suggest that central fixation declines gradually as cone density decreases rather than abruptly at a fixed cutoff. Rather than defining an abrupt biological breakpoint, this exploratory 1-mm^2^ threshold represents a practical indicator of heightened risk for fixation deterioration. Given the model's modest discrimination performance (AUC, 0.66) and lack of external validation, this threshold should be interpreted as hypothesis generation, reflecting a continuous relationship where expanding structural loss progressively increases functional vulnerability.

Longitudinally, the cumulative burden of EZ area loss, rather than short-term progression, appears to be the primary determinant of fixation stability. This suggests that the visual system can compensate until a critical scotoma size is reached, beyond which fixation control begins to fail. Future studies integrating gaze tracking and PRL heatmaps could further explore adaptive mechanisms as EZ area loss expands.

The association between baseline fixation stability and BCVA provides additional evidence that fixation stability may be clinically relevant to visual function. However, the absence of an association between baseline fixation stability and subsequent BCVA change, or between changes in fixation stability and changes in BCVA, suggests that these measures capture different aspects of visual dysfunction. This finding is consistent with the known limitations of BCVA as a marker of overall disease progression in MacTel, where EZ disruption frequently occurs in the parafoveal and temporal macula while high-contrast letter acuity primarily reflects foveal function.^24^ Fixation stability may provide complementary information regarding macular dysfunction not captured by BCVA alone.

Recent therapeutic advances underscore the clinical relevance of these findings. Revakinagene taroretcel-lwey has been shown to slow the progression of EZ area loss in MacTel.^13^ In this context, understanding how incremental EZ area loss translates into measurable functional decline becomes particularly important. Our identification of a structural threshold associated with increased risk of fixation instability provides insight into the potential functional implications of delaying EZ expansion. While our study was not designed to determine optimal treatment timing, these exploratory results provide conceptual context supporting the preservation of photoreceptor integrity before significant decline occurs.

In the parent phase 3 trials, revakinagene taroretcel-lwey significantly attenuated the decline in reading speed in NTMT-03-B but not NTMT-03-A.^13^ We therefore explored whether differences in fixation stability could account for the differing treatment effects observed between the trials. Baseline fixation stability across trials did not predict reading speed changes, nor did this relationship differ between trials. Thus, differing trial effects on reading speed are unlikely to be driven by baseline fixation stability, indicating these metrics capture complementary dimensions of visual performance.

The patient-level significance of fixation instability in MacTel remains incompletely understood. Although fixation stability reflects the ability to maintain a consistent PRL, its direct relationship with vision-related quality of life has not been established in MacTel. Importantly, in the present cohort, baseline fixation stability was not associated with subsequent change in reading speed, indicating that fixation stability should not be considered a surrogate for reading performance. Rather, fixation stability may represent a distinct dimension of central visual function that captures aspects of macular dysfunction not fully reflected by conventional measures such as visual acuity or reading speed.

Strengths of this analysis include a large sample size, high-quality imaging adjudicated by a central reading center, and inclusion of participants from two pivotal phase 3 trials. Limitations include the retrospective nature of the analysis and potential variability in microperimetry performance related to participant attention and test–retest reliability. Broader multimodal assessments and functional endpoints may provide additional insight into the mechanisms driving fixation instability. The spatial distribution of EZ loss relative to the foveal center may also influence fixation behavior and warrants further investigation. The present analysis focused on total EZ area loss and was not designed to evaluate the topographic relationship between EZ area loss and fixation stability. Future studies incorporating the location and spatial pattern of EZ disruption may provide additional insight into how specific patterns of photoreceptor degeneration influence the development and stability of the PRL. Additionally, although the use of a median baseline EZ area loss of 0.38 mm^2^ provided an objective, data-driven threshold, this stratification was performed post hoc. Results from post hoc subgroup analyses should be interpreted as hypothesis-generating, and the specific 0.38-mm^2^ cutoff is reflective of our cohort's median severity. The exact structural threshold predictive of functional failure may vary across different clinical populations of MacTel. Additionally, stratifying baseline EZ area loss into low and high groups simplifies a continuous biological process, which may overlook subtle variations near the threshold. Furthermore, while the divergence between the treatment and sham groups in the high-risk cohort was clinically marked (−3.8% vs. +0.6%), splitting the population increases the risk of type I error due to multiple statistical comparisons. Despite these limitations, the use of a median split ensured that the subgroups remained equally powered (*n* = 106 each), providing an exploratory framework to investigate the functional dilution effect.

## Conclusions

Contextualized within a robust body of prior literature, these findings strongly support EZ area loss as a structural–functional biomarker in MacTel. The exploratory ∼1-mm² threshold highlights a level below which fixation is reliably stable and above which individuals are at elevated risk for instability, underscoring the importance of careful monitoring of EZ integrity in both clinical care and therapeutic trials.
